# Enhanced invitation methods to increase uptake of NHS health checks: study protocol for a randomized controlled trial

**DOI:** 10.1186/1745-6215-15-342

**Published:** 2014-08-30

**Authors:** Alice S Forster, Caroline Burgess, Lisa McDermott, Alison J Wright, Hiten Dodhia, Mark Conner, Jane Miller, Caroline Rudisill, Victoria Cornelius, Martin C Gulliford

**Affiliations:** King’s College London, SE1 3QD London, UK; Lambeth-Southwark Public Health Directorate, SE1 2QH London, UK; University of Leeds, Leeds, LS2 9JT UK; London Borough of Lewisham, London, SE6 4RU UK; The London School of Economics and Political Science, WC2A 2AE London, UK

**Keywords:** Cardiovascular diseases, Risk assessment, Question-behavior effect, Behavioral medicine, Delivery of health care, NHS health check

## Abstract

**Background:**

NHS Health Checks is a new program for primary prevention of heart disease, stroke, diabetes, chronic kidney disease, and vascular dementia in adults aged 40 to 74 years in England. Individuals without existing cardiovascular disease or diabetes are invited for a Health Check every 5 years. Uptake among those invited is lower than anticipated.

**Method:**

The project is a three-arm randomized controlled trial to test the hypothesis that enhanced invitation methods, using the Question-Behaviour Effect (QBE), will increase uptake of NHS Health Checks compared with a standard invitation. Participants comprise individuals eligible for an NHS Health Check registered in two London boroughs. Participants are randomized into one of three arms. Group A receives the standard NHS Health Check invitation letter, information sheet, and reminder letter at 12 weeks for nonattenders. Group B receives a QBE questionnaire 1 week before receiving the standard invitation, information sheet, and reminder letter where appropriate. Group C is the same as Group B, but participants are offered a £5 retail voucher if they return the questionnaire. Participants are randomized in equal proportions, stratified by general practice. The primary outcome is uptake of NHS Health Checks 6 months after invitation from electronic health records. We will estimate the incremental health service cost per additional completed Health Check for trial groups B and C versus trial arm A, as well as evaluating the impact of the QBE questionnaire, and questionnaire plus voucher, on the socioeconomic inequality in uptake of Health Checks.

The trial includes a nested comparison of two methods for implementing allocation, one implemented manually at general practices and the other implemented automatically through the information systems used to generate invitations for the Health Check.

**Discussion:**

The research will provide evidence on whether asking individuals to complete a preliminary questionnaire, by using the QBE, is effective in increasing uptake of Health Checks and whether an incentive alters questionnaire return rates as well as uptake of Health Checks. The trial interventions can be readily translated into routine service delivery if they are shown to be cost-effective.

**Trial registration:**

Current Controlled Trials ISRCTN42856343. Date registered: 21.03.2013.

## Background

### The burden of cardiovascular disease

Cardiovascular disease (CVD), including coronary heart disease and stroke, is the leading cause of death in the UK [1]. In 2010, more than 45,000 individuals who were younger than 75 years died of CVD [1]. The prevalence of Type 2 diabetes, a risk factor for CVD, has increased [1], with more than 2.5 million living with a diagnosis of diabetes in England [2]. In 2008 through 2009, almost 2 million adults in England were registered as having chronic kidney disease [3]. CVD was estimated to cost the UK healthcare system £9 billion in 2009 [4]. The risk of CVD can be reduced through lifestyle and pharmacologic interventions tailored to individuals’ risk-factor profiles. A socioeconomic gradient in mortality exists from CVD, CHD, and stroke, with individuals from more-deprived backgrounds being at increased risk of death [1].

### The NHS Health Check

NHS Health Checks, a new cardiovascular disease primary prevention program, has been fully implemented in England since April 2011 [5]. All adults aged 40 to 74 years, without existing cardiovascular disease or diabetes, are invited for a five-yearly assessment of their risk-factor profile. The aim of the program is to identify people who are at increased risk of heart disease, stroke, diabetes, CKD, or vascular dementia so that graded intervention may be offered based on the individual’s cardiovascular risk score. The NHS Health Check program is estimated by the UK Department for Health to have the potential to prevent 2,000 deaths and 9,500 nonfatal myocardial infarctions and strokes each year [6]. The risk assessment is provided free at the point of delivery to any eligible individual, so it has the potential to reduce inequalities in CVD as long as uptake is equitable.

### Economic modeling for cost-effectiveness estimates

The Department of Health’s decision to introduce NHS Health Checks was based on health economic modeling suggesting that the program would be cost-effective [6]. It was estimated that the program would cost less than £3,000 per quality-adjusted life year; (QALY) [6]. The economic model assumed overall program uptake of 75%, informed by uptake data from the national breast-screening program. However, uptake appears to be <75%, with program data for 2013/2014 showing that 49% of people who were offered an NHS Health Check received one [7]. The few studies that have examined whether socioeconomic differences exist in uptake of NHS Health Checks have reported conflicting findings [8, 9]; however, evidence suggests a socioeconomic gradient in cervical and breast cancer screening attendance in the UK [10–12].

### Interventions to increase uptake of health checks

Jepson *et al.* [13] published a systematic review of 190 trials of interventions used to increase uptake of screening for a variety of conditions [13]. Interventions that targeted individuals and that comprised enhanced methods of inviting patients seemed to be effective at increasing screening uptake, including invitation letters, appointments, telephone calls and patient reminders. None of the interventions in this review aimed to increase uptake of a general CVD risk assessment. In a study of blood pressure screening, McDowell *et al.* [14] reported that patients who received an invitation letter were more likely to attend than were controls, and those who received a telephone invitation were more likely to attend than were those who received a letter. In a study of cholesterol testing [15], telephone invitations did not increase uptake compared with controls, nor did financial incentives, compared with controls.

Two recent studies evaluated interventions to increase uptake of CVD prevention. One Canadian trial [16] found that patients were more likely to attend if they were invited by telephone than by letter. Telephone invitations to promote uptake are difficult to implement on a large scale, as would be required for a national screening program. Another study of patients from one general practice examined the effect of sending a preliminary questionnaire before patients being invited for a health check [17]. This enhanced invitation method increased uptake, with 68.3% of the intervention group having a health check compared with 53.5% of the control group who received no intervention. The authors invoked the Question-Behaviour Effect (QBE), which suggests that asking questions about a behavior increases the likelihood that the behavior will be performed. Previous studies have shown that the QBE increases engagement in health-related behaviors, including cervical screening uptake in the UK [18, 19]. The effect is greater if individuals are asked if they would regret not attending for screening and if individuals complete and return the questionnaire [17, 19].

### Financial incentives to increase response rates to questionnaires

Strong evidence suggests that financial incentives for questionnaire return increase response rates. A systematic review, including 94 trials with a pooled total of 160,004 participants, found that the odds of returning a postal questionnaire were almost doubled if a financial incentive was offered [20]. As the Question-Behaviour Effect is greater among individuals who return a questionnaire [17], incentivizing questionnaire return may increase the size of any effect of a questionnaire on uptake of the NHS Health Check. A meta-analysis of 85,671 participants in 88 randomized trials of financial incentives to increase response rates for mailed questionnaires reported a significant increase in response rates for incentives up to the value of $5 [21].

### What is the potential impact on socioeconomic inequalities in uptake?

Death rates from coronary heart disease are highest in areas of greatest deprivation [1], so considering socioeconomic inequalities in the evaluation of any intervention to increase uptake of NHS Health Checks is important. Although evidence suggests that enhanced invitation methods, such as a QBE-based questionnaire, increase uptake of screening and performance of health-related behaviors, we do not know their impact on the NHS Health Checks, a relatively new program. The impact of the QBE on inequalities in uptake is also unknown. Individuals experiencing greater levels of deprivation may find it more difficult to convert their positive attitudes toward Health Checks, and their intentions to attend, into action. One of the possible mechanisms of action of the QBE is that it makes individuals’ attitudes more cognitively accessible and thus may increase the likelihood that individuals from deprived backgrounds attend. Alternatively, although we do not know if level of deprivation influences attitudes toward NHS Health Checks, evidence from cancer screening suggests that individuals experiencing greater levels of deprivation perceive fewer benefits and greater barriers to attending [22].

The QBE is stronger for individuals with positive attitudes toward, and intentions for, the behavior [17]; therefore the QBE may enhance inequalities in uptake, if any do exist. The offer of a financial incentive may increase questionnaire return rates only among those with already positive attitudes toward NHS Health Checks, so should result in increased uptake. However, if the offer of a financial incentive increases questionnaire return rates among those with less-positive attitudes, it is likely to have a lesser impact on uptake.

Little research examines how and if incentives influence uptake of screening differentially across deprivation [23]. The offer of a financial incentive may be most attractive for individuals who are experiencing deprivation and so may increase the strength of the QBE on Health Check uptake, particularly in individuals from deprived backgrounds. It will be important to consider inequalities in uptake in any investigation of the impact of the QBE on uptake of NHS Health Checks.

### Objectives

The aim of this research is to determine whether enhanced invitation methods, in using the QBE, lead to increased uptake of NHS Health Checks. The specific objectives of this research are as follows:

- To implement a randomized controlled trial (RCT) with individual participants who are eligible for NHS Health Checks as the unit of allocation. The trial will compare the effects of: standard invitation only; QBE questionnaire followed by standard invitation 1 week later; QBE questionnaire, with offer of retail voucher as incentive for questionnaire completion, followed by standard invitation 1 week later. The intervention effect will be evaluated by using the primary outcome of whether each individual completed the NHS Health Check within 182 days (6 months) of the standard invitation being sent.
- To estimate the incremental health service cost per additional health check completed for the QBE questionnaire, and the QBE questionnaire-plus-voucher trial arms, in comparison with the standard invitation.
- To evaluate the impact of the QBE questionnaire, and the QBE questionnaire plus voucher, on inequality in uptake of Health Checks between most- and least-deprived areas of residence based on the Indices of Multiple Deprivation (IMD 2010) score by postcode of residence.

## Methods

### Trial design

**Figure 1:**
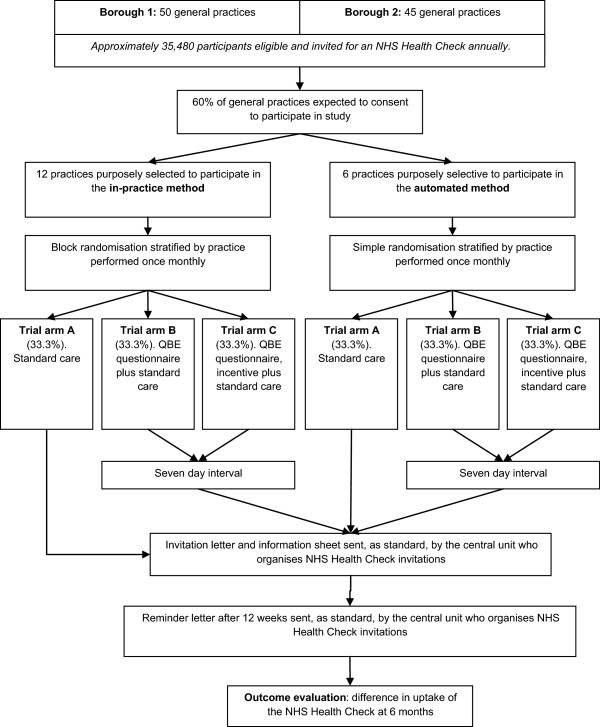
**Trial flow diagram.**

Three-arm superiority randomized controlled trial with equal allocation to each arm (see Figure 1 for the trial flow diagram).

### Setting and participants

The trial is being implemented in two London Boroughs, which are ranked 14th and 16th most-deprived local authorities in England [24]. Both boroughs are typical of areas that are in need of intervention to increase the number of individuals attending for their NHS Health Check, as uptake is below the national average (49%) [7]. In 2013/2014, 32% of individuals invited for a Health Check attended in one borough, and 38%, in the other [7].

All general practices in these boroughs are eligible and asked to consent to their patients participating in the trial. Patients are included in the trial only if their practice has provided consent. Consent to use questionnaire data is presumed, based on participants returning a questionnaire. All participants in the consented practices who are eligible to receive an NHS Health Check are included in the trial, with no exceptions from randomization or analysis.

Individuals are eligible for an NHS Health Check if they are aged between 40 and 74 years and have no existing CVD-related comorbidities and are not already prescribed antihypertensive drugs or statins. Individuals are invited for a Health Check through a cross-borough call and recall system, organized at a central unit that serves both boroughs. Each month, eligible patients are identified centrally and a pre-notification list of patients is sent to each practice for them to perform a second check for eligibility/suitability. After this, the list is considered to be ”clean”, and these patients are invited for an NHS Health Check in that month by standard invitation letter. The individual clean lists for each participating practice is the sampling frame for this trial.

### Recruitment and randomization

We are implementing two recruitment methods. For 12 purposively selected practices (from those who consent), researchers visit each practice monthly and allocate participants to each trial arm by using a pregenerated randomization list (in-practice method). Each month, the trial statistician draws up the randomization lists stratified by general practice. As all patients within a practice are assigned at one time, and to minimize the imbalance in participants between arms, random allocation uses blocks of three. The preordered randomization list is matched by the researchers to the existing order of the clean eligible patient list. For six different purposively selected practices, randomization is performed automatically by using a simple randomization procedure programmed into the information system that is used to manage the Health Check Program (automated method). For the automated method, simple randomization, stratified by practice, is being used. A randomization program has been written, and participants are automatically assigned a study ID and group allocation when the clean lists are electronically approved. Each practice is participating in the trial for a minimum of 12 months to allow seasonal variation in uptake of Health Checks.

### Interventions

Group A receives the standard invitation letter for a NHS Health Check, sent from the central unit that organizes Health Check invitations. This comprises a single-page letter from the individuals’ GPs, inviting them to make an appointment at their general practice to receive the Check or to visit a local participating pharmacy, plus an information sheet. Individuals are sent a reminder letter if they do not attend for a Health Check within 12 weeks of their first invitation.

Group B: Participants are sent the QBE questionnaire, with prepaid return envelope and covering letter 7 days before they are sent the standard NHS Health Check invitation letter and information sheet (plus reminder letter at 12 weeks, where appropriate). The QBE questionnaire is a two-page, eight-item questionnaire with one example question at the beginning. Six questions are derived from Theory of Planned Behavior [25] concepts, and two questions are based on the notion of anticipated regret. All questions are on a 7-point scale and have high readability. The questionnaire was subjected to feasibility and acceptability testing, with five individuals involved in the delivery of the NHS Health Checks program and 15 individuals from the local population in the target age range for a Health Check. Based on this feedback, the questionnaire was further refined before being used in the trial.

Group C: Participants are sent the QBE questionnaire, with prepaid return envelope and covering letter 7 days before they are sent the standard NHS Health Check invitation letter and information sheet (plus reminder letter at 12 weeks, where appropriate). The covering letter differs from that received by Group B, as participants are offered a £5 retail voucher as an incentive to return the questionnaire.

### Outcomes

The primary outcome is uptake of NHS Health Checks 182 days (6 months) after the standard invitation. Secondary outcomes include uptake of NHS Health Checks 91 days (3 months) after standard invitation and time elapsed between participants receiving the standard invitation and Health Check (among those who attend). Outcomes are extracted from general practice records by members of the research team by using nationally specified READ codes to record completion of NHS Health Checks. Participants’ postcodes are converted into an Indices of Multiple Deprivation score (IMD 2010) as a marker of deprivation.

The study received ethical approval from the NRES Committee London, London Bridge committee (13/LO/01/97).

### Sample size

It will be important to detect even modest increments in screening uptake between trial arms, because in a public health program, small effects may yield substantial benefits across the population at risk. We make the statistically conservative assumption that the underlying proportion of people invited who actually receive a Health Check is about 50%. With 4,263 participants in each trial arm, with 12,789 in total, this provides more than 90% power to detect a difference in uptake of Health Checks between each active treatment arm and the standard intervention arm of at least 4%. These calculations are based on 5% significance level by using a Bonferroni correction for three comparisons. The calculations were performed in Stata version 12 [26]. Because present rates of Health Check uptake are below 50% in the study area, slightly greater power may be realized in the study. No planned interim analysis and no stopping guidelines exist, as this trial has a low risk of adverse events.

### Blinding

Participants’ GPs have provided consent to their participation in the trial, and so participants are not overtly aware that other trial arms exist. However, participants in Groups B and C receive a postal intervention and thus cannot be blind to their group allocation. The study team is blind to participants’ details during group allocation and blind to group allocation during extraction of participant data and outcome from GP records.

### Statistical methods

The analysis will be on an intention-to-treat basis and will include all patients who are randomized, regardless of subsequent actions (for example, if the questionnaire was not sent out, participants will still be analyzed in the group to which they were randomized). For participants who die or leave their general practice within 6 months of the standard invitation, we will use the Health Check uptake status at the end of registration as their outcome.

In an individually randomized trial, Health Check uptake may vary between general practices, as, for example, if a practice nurse follows-up patients for an appointment. We will quantify the extent of between-practice variation by tabulating the outcome by general practice and estimating an intraclass correlation coefficient by using analysis of variance.

To adjust for practice-effect in the primary analysis, we will fit a marginal model by using the method of generalized estimating equations (GEEs), allowing for clustering by practice by using an exchangeable correlation structure and model-based variance estimates, as the number of practices is relatively small [27]. Covariates will be age, gender, month of invitation, deprivation quintile, and arm comparing outcomes between arms A versus B, A versus C, and B versus C, with adjustment for multiple comparisons.

The GEE model will be implemented by using the binomial family and an identity link to estimate the adjusted risk difference. Because we are primarily interested in absolute differences in NHS Health Check uptake, rather than in relative measures such as odds ratios, we will implement covariate adjustment by the method of inverse probability weighting by using the propensity score as suggested by Ukoumunne *et al*. [28]. Interaction terms will be tested, and, where appropriate, intervention effects will be estimated by subgroup. The slope index of inequality (SII) [29] will be used as a metric to estimate the difference in screening uptake between the least- and the most-deprived quintiles (local IMD scores will be classified into quintiles of the distribution of IMD scores in England for reference), and we will test whether SII differs between trial arms.

All estimates will be presented with 95% confidence intervals and *P* values. The trial will be reported according to CONSORT recommendations.

### Secondary analyses

To understand the processes of responding to the intervention, analyses will be implemented to determine whether completing the QBE questionnaire, as opposed to receiving but not completing the questionnaire, is necessary to obtain the observed effects. We will compare the proportions of patients who attend Health Checks among participants who received and completed the questionnaire for the QBE arm (completers) and the QBE + incentive arm (incentive-completers), as well as participants in the QBE arm who received but did not complete the questionnaire (noncompleters) and participants in the QBE + incentive arm who received but did not complete the questionnaire (incentive-noncompleters), and those who did not receive the questionnaire.

We will also examine whether having higher or more-positive cognitions (that is, attitude, subjective norms, perceived behavioral control, anticipated regret, intentions) about the Health Check, when completing the QBE questionnaire is associated with increased rates of attendance. Therefore, we will compare the proportions of patients who attend Health Checks for positive completers (those with high scores on the cognitions) and negative completers (those with low scores on the cognitions). If significant differences in uptake appear between the QBE and QBE + incentive arms, we will examine whether these are mediated by differences in cognitions [30].

The response rate to the QBE questionnaire will be analyzed both overall, for each treatment group, and by deprivation quintile. The distributions of responses to questionnaire items will be evaluated by deprivation quintile. We plan to access the results of the participants’ Health Checks, in terms of level of cardiovascular risk, and compare these by trial arm. We will compare Health Check results for questionnaire responders and nonresponders. These analyses will thus describe the pattern of response to the intervention in terms of both deprivation category and level of cardiovascular risk.

Secondary analyses to examine whether outcomes and intervention effects vary by deprivation quintile, gender, or age-group will be performed in a similar manner as stated earlier and will include exploring interaction effects. The delay in uptake between arms will be examined by using time-to-event analysis. The duration between the date the invitation was sent and the booked appointment will be calculated. Differences will initially be visually assessed by using Kaplan-Meier curves, and then an appropriate time-to-event model with robust variance estimates allowing clustering by practice will be fitted, adjusting for age, gender, month, and deprivation quintile.

To evaluate the consistency of estimates obtained by using the two different randomization methods, subgroup analyses will be performed for each randomization method separately. A meta-analysis will be performed, by using each participating general practice as the unit of analysis, with results pooled for each randomization method separately and across all practices. A test for heterogeneity will be performed.

### Economic evaluation

If either the enhanced invitation or the enhance invitation with incentives arms are more effective in increasing uptake than standard practice, we will analyze the cost-effectiveness of these treatments. The additional costs to the health service of an enhanced invitation and financial incentives will be estimated. Although these programs would add costs, they will be assessed against the potential long-term health benefits of NHS Health Checks by using existing models to estimate the cost-effectiveness of the Health Check program developed both nationally [6] and locally.

## Discussion

The NHS Health Check Programme is a large public health program, with nearly 16 million adults invited to participate over a 5-year period. Even small improvements in the delivery of the program have the potential to yield important benefits for public health. If the proposed interventions are effective and cost effective, then these can be rolled out across the NHS.

## Trial status

Recruitment ongoing.

## Abbreviations

95% CI: 95% confidence interval
CVD: cardiovascular disease
GEE: generalized estimating equation
GP: general practitioner
IMDs: indices of multiple deprivation
NHS: National Health Service
NIHR: National Institute for Health Research
OR: odds ratio
QBE: question-behavior effect
RCT: randomized controlled trial
RR: relative risk
SII: slope index of inequality.
